# 6,8-Di­bromo-3-nitro-2-phenyl-2*H*-chromene

**DOI:** 10.1107/S160053681301221X

**Published:** 2013-05-11

**Authors:** Lin-Jie Yan, Sheng-Yong Zhang

**Affiliations:** a Department of Medicinal Chemistry, School of Pharmacy, Fourth Military Medical University, Changle Xilu 169, 710032 Xi-An, People’s Republic of China

## Abstract

In the title compound, C_15_H_9_Br_2_NO_3_, the chromene unit is not quite planar (r.m.s. deviation from planarity = 0.0888 Å). The di­hydro­pyran ring adopts an envelope conformation with the phenyl-substituted C atom fused to the di­hydro­pyran ring as the flap. The dihedral angle between the plane defined by this C atom and the adjacent C and O atoms and the mean plane of the di­hydro­pyran ring excluding the phenyl-substituted C atom is 25.1 (3)°. The dihedral angle between the mean plane of the chromene unit and the phenyl ring is 85.7 (1)°. The crystal structure features C—H⋯O hydrogen bonds and Br⋯O contacts [3.289 (3) Å] involving the nitro O atoms.

## Related literature

For the preparation of analogs of the title compound, see: Yan *et al.* (2001 ▶); Pateliya *et al.* (2009 ▶). For synthetic uses of the analogs and bioactive derivatives of the title compound, see: Furuta *et al.* (2007 ▶); Pateliya *et al.* (2009 ▶).
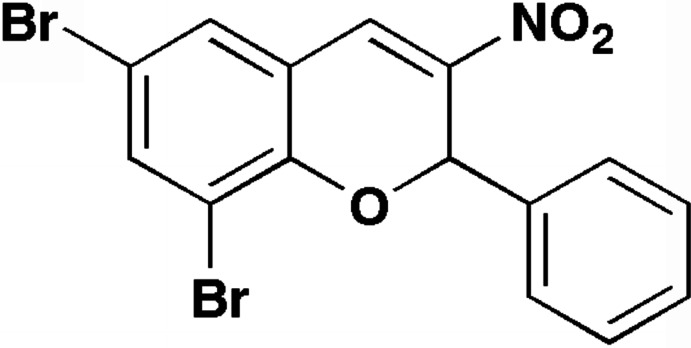


## Experimental

### 

#### Crystal data

C_15_H_9_Br_2_NO_3_
*M* *_r_* = 411.05
Triclinic, 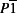

*a* = 8.2249 (19) Å
*b* = 8.886 (2) Å
*c* = 10.814 (3) Å
α = 73.503 (4)°
β = 75.633 (4)°
γ = 79.579 (4)°
*V* = 728.8 (3) Å^3^
*Z* = 2
Mo *K*α radiation
μ = 5.57 mm^−1^
*T* = 296 K
0.37 × 0.24 × 0.14 mm

#### Data collection

Bruker APEXII CCD diffractometer
Absorption correction: multi-scan (*SADABS*; Bruker, 2005 ▶) *T*_min_ = 0.232, *T*_max_ = 0.505
3687 measured reflections
2563 independent reflections
1856 reflections with *I* > 2σ(*I*)
*R*_int_ = 0.029

#### Refinement

*R*[*F* ^2^ > 2σ(*F* ^2^)] = 0.041
*wR*(*F* ^2^) = 0.105
*S* = 1.04
2563 reflections
190 parameters
H-atom parameters constrained
Δρ_max_ = 0.54 e Å^−3^
Δρ_min_ = −0.56 e Å^−3^

### 

Data collection: *APEX2* (Bruker, 2008 ▶); cell refinement: *SAINT* (Bruker, 2008 ▶); data reduction: *SAINT*; program(s) used to solve structure: *SHELXS97* (Sheldrick, 2008 ▶); program(s) used to refine structure: *SHELXL97* (Sheldrick, 2008 ▶); molecular graphics: *SHELXTL* (Sheldrick, 2008 ▶); software used to prepare material for publication: *SHELXTL*.

## Supplementary Material

Crystal structure: contains datablock(s) I, global. DOI: 10.1107/S160053681301221X/zl2545sup1.cif

Structure factors: contains datablock(s) I. DOI: 10.1107/S160053681301221X/zl2545Isup2.hkl

Supplementary material file. DOI: 10.1107/S160053681301221X/zl2545Isup3.cml

Additional supplementary materials:  crystallographic information; 3D view; checkCIF report

## Figures and Tables

**Table 1:** Hydrogen-bond geometry (Å, °)

*D*—H⋯*A*	*D*—H	H⋯*A*	*D*⋯*A*	*D*—H⋯*A*
C5—H5⋯O2^i^	0.93	2.61	3.404	144
C7—H7⋯O2^i^	0.93	2.47	3.265	143

Symmetry code: (i) 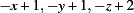.

**Fig. 1.**
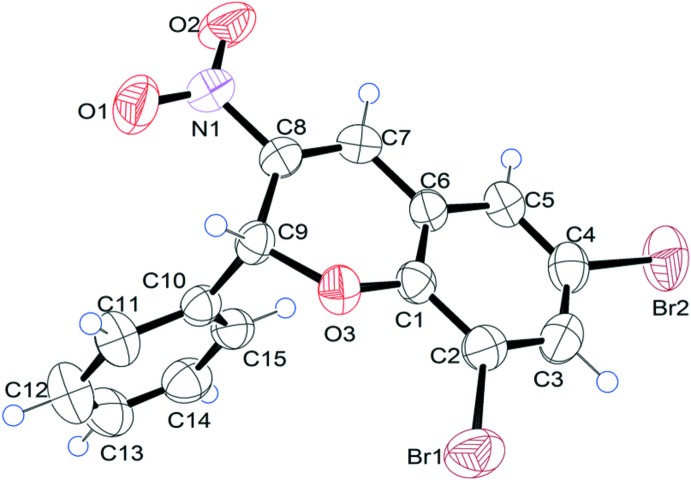
Figure 1 The molecular structure of the title compound, showing the atom-labelling scheme. Displacement ellipsoids are drawn at the 50% probability level.

**Fig. 2.**
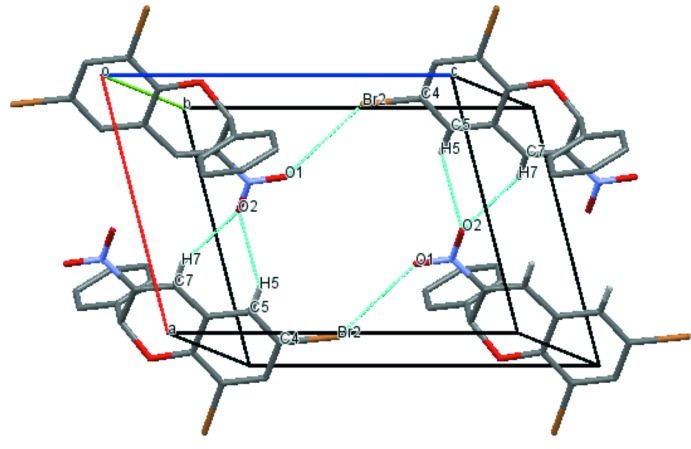
Figure 2 Crystal packing in the title compound where molecules are linked via C–H···O hydrogen bonds and C—Br···O bond (dashed lines). Except for those involved in hydrogen-bonding interactions, H atoms have been omitted for clarity.

## supplementary crystallographic information

### Comment

3-Nitro-2*H*-chromene derivatives are important intermediates for synthesis of (-)-epigallocatechin gallate (EGCG) which is a promising candidate for drug development for its cancer preventive, antiviral, and other important bioactivities and is a major constituent of green tea extract (Furuta *et al.*, 2007). In addition, 3-nitro-2-phenyl-2*H*-chromene derivatives were used for synthesis of the 1, 2, 3-triazole heterocycles which are reported to posses several biological activities, including anti-HIV, anti-allergic, anti-fungal, anti-viral, and anti-microbial (Pateliya *et al.*, 2009). The title compound, oxa-6,8-dibromo-3-nitro-2-phenyl-2*H*-chromene, is such a 3-nitro-2*H*-chromene derivative. It has been synthesized by tandem oxa-Henry-Michael reaction of 4,5-dibromo-2-hydroxy-benzaldehyde with (2-nitro-vinyl)-benzene (Yan *et al.*, 2001).

In the title compound, C_15_H_9_Br_2_NO_3_, the chromene unit is not quite planar. The r.m.s. deviation from planarity is 0.0888 Å (fitted atoms are C1, C2, C3, C4, C5, C6, C7, C8, C9 and O3). The dihydropyran ring adopts an envelope conformation (with atom C9 as the flap). The dihedral angle between the plane defined by C8, C9 and O3 and the least-squares plane defined by C1, C6, C7, C8 and O3 is 25.1 (3)°. The dihedral angle between the phenyl ring and the overall average plane of the chromene unit (defined by atoms C1, C2, C3, C4, C5, C6, C7, C8, C9 and O3) is 85.7 (1)°. The structure is stabilized by intermolecular C5—H5···O2 and C7—H7···O2 hydrogen bonds and a C4—Br2···O1 halogen bond (Fig. 2), with the nitro O atoms acceptors for the C—H and C—Br groups.

### Experimental

In a 100 ml Schlenk tube were placed 4,5-dibromo-2-hydroxy-benzaldehyde (2.26 g, 5.5 mmol), (2-nitro-vinyl)-benzene (0.75 g, 5 mmol), and 1,4-diazabicyclo[2.2.2]octane (DABCO) (0.56 g, 5 mmol), and the flask was evacuated and filled with N_2_ three times, anhydrous toluene (20 ml, degassed) was added to the tube. The reaction mixture was stirred for 1.5 h at 80 °C under N_2_. The solvent was removed under reduced pressure to give the crude product as a brown oil. The crude product was purified by a flash column chromatography with the elution of *n*-hexane/ethyl acetate (20/1) to yield the title compound (*R*_f_ = 0.65) as a light yellow powder (76%). ^1^H NMR (CDCl_3_, 500 MHz): δ 8.15 (s, 1H), 7.50 (s, 1H), 7.35–7.32 (m, 2H), 7.1 (s, 2H), 7.04–6.99 (m, 2H), 6.8 (d, 1H).

Single crystals of the compound were obtained from dry CH_2_Cl_2_.

### Refinement

All H atoms were placed in calculated positions and refined as riding, with C—H distances of 0.93 Å and 0.98 Å, and with *U*_iso_(H) = 1.2*U*_eq_(C) for aromatic and methylidyne H atoms.

### Crystal data

**Table:**

C_15_H_9_Br_2_NO_3_	*F*(000) = 400
*M**_r_* = 411.05	*D*_x_ = 1.873 Mg m^−^^3^
Triclinic, *P*1	Melting point: 358 K
*a* = 8.2249 (19) Å	Mo *K*α radiation, λ = 0.71073 Å
*b* = 8.886 (2) Å	Cell parameters from 1256 reflections
*c* = 10.814 (3) Å	θ = 2.6–23.4°
α = 73.503 (4)°	µ = 5.57 mm^−^^1^
β = 75.633 (4)°	*T* = 296 K
γ = 79.579 (4)°	Block, yellow
*V* = 728.8 (3) Å^3^	0.37 × 0.24 × 0.14 mm
*Z* = 2	

### Data collection

**Table:**

Bruker APEXII CCD diffractometer	2563 independent reflections
Radiation source: fine-focus sealed tube	1856 reflections with *I* > 2σ(*I*)
Graphite monochromator	*R*_int_ = 0.029
φ and ω scans	θ_max_ = 25.1°, θ_min_ = 2.4°
Absorption correction: multi-scan (*SADABS*; Bruker, 2005)	*h* = −9→9
*T*_min_ = 0.232, *T*_max_ = 0.505	*k* = −10→8
3687 measured reflections	*l* = −12→9

### Refinement

**Table:**

Refinement on *F*^2^	Primary atom site location: structure-invariant direct methods
Least-squares matrix: full	Secondary atom site location: difference Fourier map
*R*[*F*^2^ > 2σ(*F*^2^)] = 0.041	Hydrogen site location: inferred from neighbouring sites
*wR*(*F*^2^) = 0.105	H-atom parameters constrained
*S* = 1.04	*w* = 1/[σ^2^(*F*_o_^2^) + (0.0459*P*)^2^] where *P* = (*F*_o_^2^ + 2*F*_c_^2^)/3
2563 reflections	(Δ/σ)_max_ = 0.001
190 parameters	Δρ_max_ = 0.54 e Å^−^^3^
0 restraints	Δρ_min_ = −0.56 e Å^−^^3^

### Special details

**Table:**

Geometry. All e.s.d.'s (except the e.s.d. in the dihedral angle between two l.s. planes) are estimated using the full covariance matrix. The cell e.s.d.'s are taken into account individually in the estimation of e.s.d.'s in distances, angles and torsion angles; correlations between e.s.d.'s in cell parameters are only used when they are defined by crystal symmetry. An approximate (isotropic) treatment of cell e.s.d.'s is used for estimating e.s.d.'s involving l.s. planes.
Refinement. Refinement of *F*^2^ against ALL reflections. The weighted *R*-factor *wR* and goodness of fit *S* are based on *F*^2^, conventional *R*-factors *R* are based on *F*, with *F* set to zero for negative *F*^2^. The threshold expression of *F*^2^ > σ(*F*^2^) is used only for calculating *R*-factors(gt) *etc*. and is not relevant to the choice of reflections for refinement. *R*-factors based on *F*^2^ are statistically about twice as large as those based on *F*, and *R*- factors based on ALL data will be even larger.

### Fractional atomic coordinates and isotropic or equivalent isotropic displacement parameters (Å^2^)

**Table:**

	*x*	*y*	*z*	*U*_iso_*/*U*_eq_	
Br1	1.27050 (6)	0.90953 (6)	0.84090 (5)	0.0643 (2)	
Br2	0.93397 (8)	0.62857 (8)	1.35367 (5)	0.0800 (2)	
N1	0.6350 (4)	0.6139 (4)	0.7380 (4)	0.0480 (9)	
O1	0.6475 (4)	0.6655 (5)	0.6195 (3)	0.0686 (10)	
O2	0.5354 (4)	0.5232 (4)	0.8073 (3)	0.0699 (10)	
O3	1.0007 (3)	0.7836 (3)	0.7672 (3)	0.0431 (7)	
C1	0.9785 (5)	0.7523 (5)	0.8998 (4)	0.0377 (9)	
C2	1.0929 (5)	0.7996 (5)	0.9515 (4)	0.0441 (10)	
C3	1.0787 (5)	0.7621 (5)	1.0868 (4)	0.0521 (12)	
H3	1.1558	0.7935	1.1216	0.063*	
C4	0.9514 (5)	0.6788 (5)	1.1700 (4)	0.0494 (11)	
C5	0.8386 (5)	0.6283 (5)	1.1199 (4)	0.0449 (10)	
H5	0.7545	0.5694	1.1763	0.054*	
C6	0.8516 (5)	0.6659 (5)	0.9847 (4)	0.0390 (10)	
C7	0.7392 (5)	0.6132 (5)	0.9260 (4)	0.0409 (10)	
H7	0.6635	0.5423	0.9776	0.049*	
C8	0.7458 (5)	0.6672 (5)	0.7984 (4)	0.0382 (9)	
C9	0.8570 (5)	0.7858 (5)	0.7090 (4)	0.0375 (9)	
H9	0.9015	0.7541	0.6261	0.045*	
C10	0.7659 (5)	0.9503 (5)	0.6771 (4)	0.0396 (10)	
C11	0.7715 (6)	1.0345 (6)	0.5490 (5)	0.0570 (12)	
H11	0.8330	0.9893	0.4812	0.068*	
C12	0.6882 (7)	1.1838 (7)	0.5188 (5)	0.0702 (15)	
H12	0.6930	1.2391	0.4311	0.084*	
C13	0.5985 (6)	1.2509 (6)	0.6174 (6)	0.0680 (15)	
H13	0.5422	1.3526	0.5972	0.082*	
C14	0.5908 (6)	1.1686 (6)	0.7473 (5)	0.0576 (13)	
H14	0.5293	1.2144	0.8148	0.069*	
C15	0.6736 (5)	1.0198 (5)	0.7763 (4)	0.0447 (10)	
H15	0.6680	0.9643	0.8640	0.054*	

### Atomic displacement parameters (Å^2^)

**Table:**

	*U*^11^	*U*^22^	*U*^33^	*U*^12^	*U*^13^	*U*^23^
Br1	0.0462 (3)	0.0659 (4)	0.0820 (4)	−0.0181 (2)	−0.0097 (2)	−0.0162 (3)
Br2	0.0990 (4)	0.0991 (5)	0.0454 (3)	0.0001 (4)	−0.0277 (3)	−0.0197 (3)
N1	0.046 (2)	0.050 (2)	0.052 (3)	−0.0092 (18)	−0.0085 (19)	−0.020 (2)
O1	0.076 (2)	0.092 (3)	0.046 (2)	−0.024 (2)	−0.0183 (18)	−0.0163 (19)
O2	0.071 (2)	0.085 (3)	0.066 (2)	−0.045 (2)	−0.0055 (18)	−0.023 (2)
O3	0.0343 (14)	0.0536 (18)	0.0381 (17)	−0.0051 (13)	−0.0062 (12)	−0.0077 (14)
C1	0.034 (2)	0.037 (2)	0.041 (3)	0.0003 (18)	−0.0100 (18)	−0.0078 (19)
C2	0.036 (2)	0.041 (2)	0.056 (3)	0.0031 (19)	−0.012 (2)	−0.015 (2)
C3	0.050 (3)	0.056 (3)	0.059 (3)	0.003 (2)	−0.022 (2)	−0.024 (3)
C4	0.050 (3)	0.053 (3)	0.044 (3)	0.007 (2)	−0.014 (2)	−0.016 (2)
C5	0.043 (2)	0.045 (3)	0.041 (3)	−0.001 (2)	−0.008 (2)	−0.006 (2)
C6	0.041 (2)	0.038 (2)	0.038 (2)	−0.0024 (18)	−0.0117 (19)	−0.0065 (19)
C7	0.040 (2)	0.036 (2)	0.045 (3)	−0.0062 (18)	−0.0043 (19)	−0.011 (2)
C8	0.036 (2)	0.040 (2)	0.040 (3)	−0.0041 (18)	−0.0053 (19)	−0.015 (2)
C9	0.037 (2)	0.046 (3)	0.032 (2)	−0.0069 (18)	−0.0066 (18)	−0.0113 (19)
C10	0.036 (2)	0.044 (2)	0.041 (3)	−0.0084 (19)	−0.0094 (19)	−0.010 (2)
C11	0.060 (3)	0.060 (3)	0.043 (3)	−0.002 (2)	−0.010 (2)	−0.005 (2)
C12	0.076 (3)	0.070 (4)	0.053 (3)	0.004 (3)	−0.019 (3)	−0.001 (3)
C13	0.063 (3)	0.046 (3)	0.090 (5)	0.003 (3)	−0.032 (3)	−0.001 (3)
C14	0.055 (3)	0.050 (3)	0.073 (4)	−0.001 (2)	−0.016 (3)	−0.024 (3)
C15	0.044 (2)	0.043 (3)	0.048 (3)	−0.007 (2)	−0.010 (2)	−0.011 (2)

### Geometric parameters (Å, º)

**Table:**

Br1—C2	1.872 (4)	C7—C8	1.316 (5)
Br2—C4	1.882 (4)	C7—H7	0.9300
N1—O2	1.214 (4)	C8—C9	1.487 (6)
N1—O1	1.217 (4)	C9—C10	1.504 (5)
N1—C8	1.453 (5)	C9—H9	0.9800
O3—C1	1.352 (5)	C10—C11	1.369 (6)
O3—C9	1.465 (4)	C10—C15	1.382 (6)
C1—C2	1.384 (6)	C11—C12	1.368 (7)
C1—C6	1.391 (6)	C11—H11	0.9300
C2—C3	1.385 (6)	C12—C13	1.360 (7)
C3—C4	1.373 (6)	C12—H12	0.9300
C3—H3	0.9300	C13—C14	1.380 (6)
C4—C5	1.376 (6)	C13—H13	0.9300
C5—C6	1.385 (5)	C14—C15	1.362 (6)
C5—H5	0.9300	C14—H14	0.9300
C6—C7	1.447 (6)	C15—H15	0.9300
			
O2—N1—O1	124.0 (4)	C7—C8—C9	124.5 (4)
O2—N1—C8	119.0 (4)	N1—C8—C9	116.0 (4)
O1—N1—C8	116.9 (4)	O3—C9—C8	109.5 (3)
C1—O3—C9	119.2 (3)	O3—C9—C10	110.4 (3)
O3—C1—C2	118.3 (4)	C8—C9—C10	113.3 (3)
O3—C1—C6	122.2 (3)	O3—C9—H9	107.8
C2—C1—C6	119.4 (4)	C8—C9—H9	107.8
C1—C2—C3	119.7 (4)	C10—C9—H9	107.8
C1—C2—Br1	120.9 (3)	C11—C10—C15	118.4 (4)
C3—C2—Br1	119.4 (3)	C11—C10—C9	120.7 (4)
C4—C3—C2	120.5 (4)	C15—C10—C9	120.9 (4)
C4—C3—H3	119.7	C10—C11—C12	121.3 (5)
C2—C3—H3	119.7	C10—C11—H11	119.4
C3—C4—C5	120.5 (4)	C12—C11—H11	119.4
C3—C4—Br2	120.0 (3)	C13—C12—C11	119.7 (5)
C5—C4—Br2	119.6 (4)	C13—C12—H12	120.1
C4—C5—C6	119.4 (4)	C11—C12—H12	120.1
C4—C5—H5	120.3	C12—C13—C14	120.1 (5)
C6—C5—H5	120.3	C12—C13—H13	119.9
C5—C6—C1	120.6 (4)	C14—C13—H13	119.9
C5—C6—C7	121.9 (4)	C15—C14—C13	119.7 (5)
C1—C6—C7	117.4 (4)	C15—C14—H14	120.2
C8—C7—C6	118.9 (4)	C13—C14—H14	120.2
C8—C7—H7	120.6	C14—C15—C10	120.8 (4)
C6—C7—H7	120.6	C14—C15—H15	119.6
C7—C8—N1	119.4 (4)	C10—C15—H15	119.6
			
C9—O3—C1—C2	−159.3 (3)	O2—N1—C8—C7	1.8 (6)
C9—O3—C1—C6	24.9 (5)	O1—N1—C8—C7	−179.0 (4)
O3—C1—C2—C3	−176.7 (3)	O2—N1—C8—C9	−176.3 (3)
C6—C1—C2—C3	−0.7 (6)	O1—N1—C8—C9	2.8 (5)
O3—C1—C2—Br1	1.8 (5)	C1—O3—C9—C8	−33.4 (5)
C6—C1—C2—Br1	177.7 (3)	C1—O3—C9—C10	92.0 (4)
C1—C2—C3—C4	−0.3 (6)	C7—C8—C9—O3	23.2 (5)
Br1—C2—C3—C4	−178.7 (3)	N1—C8—C9—O3	−158.8 (3)
C2—C3—C4—C5	1.5 (6)	C7—C8—C9—C10	−100.6 (4)
C2—C3—C4—Br2	179.9 (3)	N1—C8—C9—C10	77.5 (4)
C3—C4—C5—C6	−1.8 (6)	O3—C9—C10—C11	110.8 (4)
Br2—C4—C5—C6	179.8 (3)	C8—C9—C10—C11	−126.0 (4)
C4—C5—C6—C1	0.8 (6)	O3—C9—C10—C15	−69.7 (5)
C4—C5—C6—C7	178.6 (4)	C8—C9—C10—C15	53.5 (5)
O3—C1—C6—C5	176.3 (3)	C15—C10—C11—C12	0.0 (7)
C2—C1—C6—C5	0.4 (6)	C9—C10—C11—C12	179.5 (5)
O3—C1—C6—C7	−1.6 (5)	C10—C11—C12—C13	0.2 (8)
C2—C1—C6—C7	−177.4 (4)	C11—C12—C13—C14	−0.3 (8)
C5—C6—C7—C8	172.6 (4)	C12—C13—C14—C15	0.1 (7)
C1—C6—C7—C8	−9.6 (6)	C13—C14—C15—C10	0.2 (7)
C6—C7—C8—N1	179.3 (3)	C11—C10—C15—C14	−0.3 (6)
C6—C7—C8—C9	−2.7 (6)	C9—C10—C15—C14	−179.8 (4)

### Hydrogen-bond geometry (Å, º)

**Table:**

*D*—H···*A*	*D*—H	H···*A*	*D*···*A*	*D*—H···*A*
C5—H5···O2^i^	0.93	2.61	3.404	144
C7—H7···O2^i^	0.93	2.47	3.265	143

Symmetry code: (i) −*x*+1, −*y*+1, −*z*+2.

## References

[bb1] Bruker (2005). *SADABS* Bruker AXS Inc., Madison, Wisconsin, USA.

[bb2] Bruker (2008). *APEX2* and *SAINT* Bruker AXS Inc., Madison, Wisconsin, USA.

[bb3] Furuta, T., Hirooka, Y., Abe, A., Sugata, Y., Ueda, M., Murakami, K., Suzuki, T., Tanaka, K. & Kan, T. (2007). *Bioorg. Med. Chem. Lett.* **17**, 3095–3098. 10.1016/j.bmcl.2007.03.041 17420124

[bb4] Pateliya, M. H., Rama Raju, B., Kavala, V., Kuo, C.-W. & Yao, C.-F. (2009). *Tetrahedron*, **65**, 5799–5804.

[bb5] Sheldrick, G. M. (2008). *Acta Cryst.* A**64**, 112–122. 10.1107/S0108767307043930 18156677

[bb6] Yan, M. C., Jang, Y. J. & Yao, C. F. (2001). *Tetrahedron Lett.* **42**, 2717–2721.

